# Impaired neutralizing antibody response to COVID-19 mRNA vaccines in cancer patients

**DOI:** 10.1186/s13578-021-00713-2

**Published:** 2021-11-21

**Authors:** Cong Zeng, John P. Evans, Sarah Reisinger, Jennifer Woyach, Christina Liscynesky, Zeinab El Boghdadly, Mark P. Rubinstein, Karthik Chakravarthy, Linda Saif, Eugene M. Oltz, Richard J. Gumina, Peter G. Shields, Zihai Li, Shan-Lu Liu

**Affiliations:** 1 Center for Retrovirus Research, The Ohio State University, Columbus, OH 43210 USA; 2 Department of Veterinary Biosciences, The Ohio State University, Columbus, OH 43210 USA; 3 Molecular, Cellular and Developmental Biology Program, The Ohio State University, 43210 Columbus, OH USA; 4 Comprehensive Cancer Center, James Cancer Hospital, The Ohio State University, Columbus, OH 43210 USA; 5 Division of Hematology, James Comprehensive Cancer Center, The Ohio State University, Columbus, OH 43210 USA; 6 Internal Medicine, Division of Infectious Diseases, Department of Internal Medicine, The Ohio State University, Columbus, OH 43210 USA; 7 Division of Medical Oncology, Department of Internal Medicine, Pelotonia Institute for Immuno-Oncology, The Ohio State University Comprehensive Cancer Center, Columbus, OH 43210 USA; 8 Center for Food Animal Health, Animal Sciences Department, OARDC, College of Food, Agricultural and Environmental Sciences,, Wooster, OH 44691 USA; 9 Veterinary Preventive Medicine Department, College of Veterinary Medicine, The Ohio State University, 44691 Wooster, OH USA; 10 Viruses and Emerging Pathogens Program, Infectious Diseases Institute, The Ohio State University, Columbus, OH 43210 USA; 11 Department of Microbial Infection and Immunity, The Ohio State University, 43210 Columbus, OH USA; 12 Department of Medicine, The Ohio State University, Columbus, OH 43210 USA

## Abstract

There is currently a critical need to determine the efficacy of SARS-CoV-2 vaccination for immunocompromised patients. In this study, we determined the neutralizing antibody response in 160 cancer patients diagnosed with chronic lymphocytic leukemia (CLL), lung cancer, breast cancer, and various non-Hodgkin’s lymphomas (NHL), after they received two doses of mRNA vaccines. Serum from 46 mRNA vaccinated health care workers (HCWs) served as healthy controls. We discovered that (1) cancer patients exhibited reduced neutralizing antibody titer (NT_50_) compared to HCWs; (2) CLL and NHL patients exhibited the lowest NT_50_ levels, with 50-60% of them below the detection limit; (3) mean NT_50_ levels in patients with CLL and NHL was ~2.6 fold lower than those with solid tumors; and (4) cancer patients who received anti-B cell therapy exhibited significantly reduced NT_50_ levels. Our results demonstrate an urgent need for novel immunization strategies for cancer patients against SARS-CoV-2, particularly those with hematological cancers and those on anti-B cell therapies.

**Supplementary Information:**

The online version contains supplementary material available at 10.1186/s13578-021-00713-2.

## Dear Editor,

In response to the global public health crisis caused by the COVID-19 pandemic, several SARS-CoV-2 vaccines were rapidly developed including the Pfizer/BioNTech BNT162b2 and Moderna mRNA-1273 mRNA vaccines. However, clinical trials of these mRNA vaccines did not investigate their efficacy in vulnerable populations, including immunocompromised patients. With rising vaccination rates and an easing of public health measures, there is a critical need to determine the efficacy of SARS-CoV-2 vaccination for such patients, who may experience a reduced efficacy of administered vaccines [1]. It has already been demonstrated that organ transplant recipients, who are under immunosuppressive therapy to prevent rejection, exhibit reduced responsiveness to SARS-CoV-2 vaccination [2]. Cancer patients represent another critical population of immunocompromised individuals who, due to the nature of the disease or to treatment with immunomodulatory therapies, may not exhibit a robust response to mRNA vaccination. A better understanding of the factors governing response to vaccination in cancer patients is critical to inform clinical decisions about the need for booster doses, the timing of vaccine administration, the need to interrupt treatment courses for vaccination, and general guidance about the level of protection achieved by vaccination in cancer patients. To this end, this study examines the neutralizing antibody response to Pfizer/BioNTech BNT162b2 and Moderna mRNA-1273 vaccination in a cohort of patients with solid tumor and hematological malignancies.

The study population included 160 cancer patients (54 chronic lymphocytic leukemia (CLL), 45 non-Hodgkin’s lymphoma (NHL), 29 lung cancer, 30 breast cancer, and 2 breast cancer with CLL) recruited through medical record screening for vaccine appointments or recent post-vaccine administration, as well as an independent cohort of 46 health care workers (HCWs), who have no history of cancer. Cancer patients had a median age of 66 years while the median age of HCWs was 38 years. No cancer patient or HCW was COVID-19 positive as confirmed by nucleocapsid-based ELISA. About 61% of cancer patients (n = 98) and 52% of the HCWs (n = 24) received BNT162b2, compared to 39% (n = 62) and 48% (n = 22) who received the mRNA-1273, respectively. We collected serum samples for 159/160 cancer patients between 31 and 232 days (median 134 days) post-second dose, and HCW serum samples were obtained at 6 months post-second dose. Cancer diagnoses and treatments of the patients are shown in Table 1. The largest treatment groups were 47 patients with B-cell malignancies (28 CLL and 19 NHL) who received B cell depletion therapy or other B cell-suppressing drugs (such as anti-CD20 monoclonal antibodies and Bruton tyrosine kinase (BTK) inhibitors) during the study period; and 46% (n = 28) of solid tumor patients received immune checkpoint inhibitors against PD-1 or PD-L1.

**Table 1:** Demographic information of cancer patients

	Total (n = 160)		Male (n = 85)		Female (n = 75)	
	n	(%)	n	(%)	n	(%)
Age Group (years)						
30–44	11	6.9	1	1.2	10	13.3
45–59	35	21.9	12	14.1	23	30.7
60–74	96	60.0	58	68.2	38	50.7
75–85	18	11.3	14	16.5	4	5.3
Race						
African American/Black	6	3.8	2	2.4	4	5.3
Asian Chinese	3	1.9	2	2.4	1	1.3
Asian Japanese/White	1	0.6	1	1.2	0	0.0
Other	2	1.3	1	1.2	1	1.3
White	148	92.5	79	92.9	69	92.0
Vaccine Type						
Moderna	62	38.8	34	40.0	28	37.3
Pfizer	98	61.3	51	60.0	47	62.7
Cancer type						
CLL	54	33.8	40	47.1	14	18.7
Lung	29	18.1	18	21.2	11	14.7
Breast	30	18.8	0	0.0	30	40.0
CLL/Breast	2	1.3	0	0.0	2	2.7
Non–Hodgkin’s Lymphoma	45	28.1	27	31.8	18	24.0
Anti–B cell therapy						
CLL	28	17.5	23	27.1	5	6.7
Non–Hodgkin’s Lymphoma	19	11.9	11	12.9	8	10.7
Anti–PD–1/PD–L1						
Lung	26	16.3	17	20.0	9	12.0
Breast	2	1.3	0	0.0	2	2.7

The anti-B cell therapy drugs include Obinutuzumab, Rituximab, Ibrutinib, Zanubrutinib, Pirtobrutinib and Acalabrutinib

The anti-PD-1/PD-L1 drugs include Nivolumab, Pembrolizumab, Durvalumab and Atezolizumab

We assessed sera for neutralizing antibody titers using a secreted *Gaussia*-luciferase SARS-CoV-2-pseudotyped-lentivirus neutralization assay as previously described (Additional file 1) [3]. Briefly, pseudotyped virus was incubated with serial dilutions of patient sera and used to infect HEK293T-ACE2 cells (BEI NR-52,511). Infected cells then secreted *Gaussia*-luciferase into the culture media which was harvested 48 and 72 h after infection, and luminescence was measured by a BioTek Cytation5 plate-reader. The resulting luciferase output was used to calculate a neutralization titer at 50% efficiency of maximal inhibition (NT_50_). To ensure valid comparisons, the serum samples of all cancer patients and HCWs were processed side-by-side in the same experiment.

**Fig. 1:**
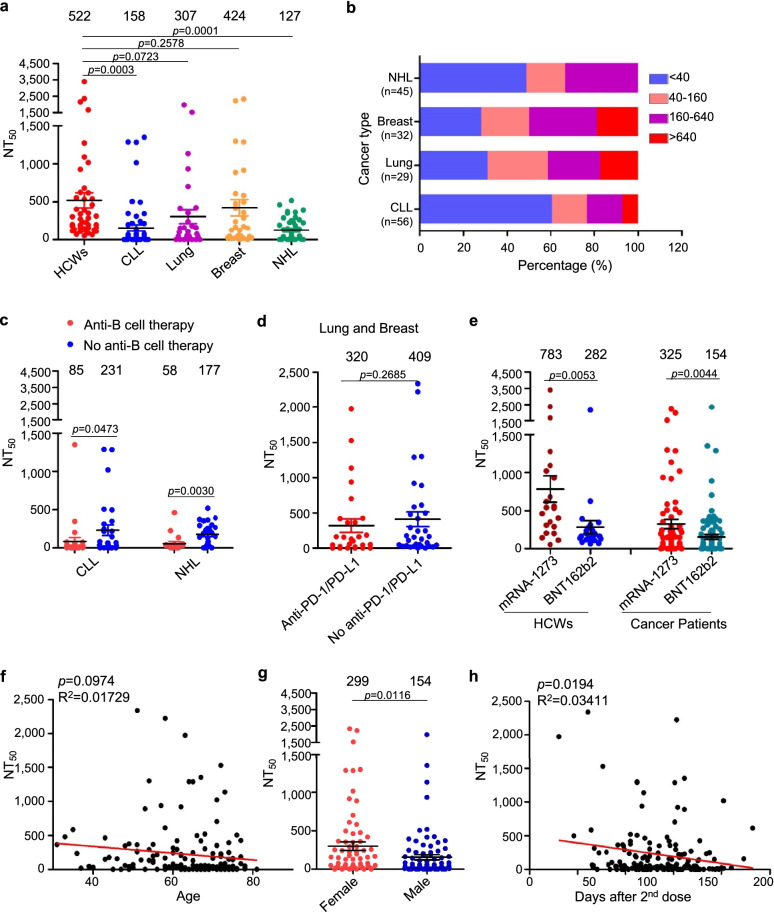
Neutralization of SARS-CoV-2 spike-pseudotyped lentivirus by sera of cancer patients and health care workers. **a** Comparison of 50% neutralization titer (NT_50_) between cancer patients and health care workers (HCWs). Serially diluted sera were incubated with SARS-CoV-2 spike-pseudotyped lentivirus, followed by infection of HEK293T-ACE2 cells. The assay was carried out side by side for samples of healthy individuals and cancer patients to ensure valid comparisons. **b** Distribution ranges of NT_50_ among four cancer patient groups. Note that 2 patients who had both CLL and breast cancer were included in each group. **c** Comparison of NT_50_ between anti-B cell therapy and no anti-B cell therapy in cancer patients. Twenty-eight out of the 54 CLL patients and 19 out of the 45 NHL patients received anti-B cell therapy, with drugs including BTK inhibitors and anti-CD20 monoclonal antibodies. **d** Comparison of NT_50_ between anti-PD1/PD-L1 and no anti-PD1/PD-L1 treatment in lung and breast cancer patients. **e** Comparison of NT_50_ between Moderna and Pfizer vaccinees in health care workers (HCWs) and cancer patients. **f** Correlative analysis between NT_50_ values and ages of cancer patients. **g** Comparison of NT_50_ values between male and female cancer patients. **h** Correlative analysis between NT_50_ values and days of collection after the second dose of vaccination. All correlative analyses were performed using Prism 5 (f and h). In all cases, NT_50_ values indicated at top were calculated by taking the inverse of the 50% inhibitory dilution values obtained from least squares regression non-linear curve modeled with Prism. Statistical significance was determined by a one-tailed unpaired t-test. CLL: Chronic Lymphocytic Leukemia; NHL: Non-Hodgkin’s Lymphoma

We first compared the neutralizing antibody titers of cancer patients with those of HCWs. Overall, cancer patients exhibited reduced neutralizing antibody responses, with a mean NT_50_ of 220 compared to a mean NT_50_ of 522 for HCWs (Fig. 1a); this is despite the relatively shorter median time (134 days) after the second dose of vaccination for cancer patients as compared to HCWs, which is an average of ~180 days. Patients with CLL exhibited the lowest neutralizing antibody response, with over 61% (n = 34) of patients exhibiting undetectable NT_50_ values (below 40), compared to 49%, 31%, and 28% for NHL (n = 22), lung cancer (n = 9), and breast cancer patients (n = 9), respectively (Fig. 1b). The mean NT_50_ of patients with CLL and NHL (158 and 127, respectively) was ~2.6 fold lower than that of solid tumor patients (369) (Fig. 1a). Interestingly, there were a few CLL patients that exhibited high titer while none were observed for the NHL patients (Fig. 1b).

Given the common usage of B-cell depleting therapies in the treatment of hematological cancers and their likelihood of impacting vaccine efficacy, we then examined the effect of anti-B-cell therapy on neutralizing antibody response. The treatment included anti-CD20 antibodies Obinutuzumab and Rituximab, as well as BTK inhibitors Ibrutinib, Zanubrutinib, Pirtobrutinib, and Acalabrutinib. Notably, we found that CLL and NHL patients who received anti-B cell therapy exhibited 2.7-fold (p = 0.0483) and 3.1-fold (p = 0.0030) reduced neutralizing antibody response to mRNA vaccine compared to those without anti-B cell therapy, respectively (Fig. 1c).

The programmed death-1 (PD-1) receptor is an important immune checkpoint molecule that promotes exhaustion/dysfunction in chronically activated T-cells. Disruption of PD-1 or its ligand PD-L1 is a common treatment to rejuvenate T cell function in cancer patients [4]. Given this role, we examined how anti-PD-1/PD-L1 treatments might modulate the host immune response to mRNA vaccination. However, we did not find significant differences in NT_50_ or development of immune-related adverse events between anti-PD-1/PD-L1 antibody-treated and un-treated lung/breast cancer patients (Fig. 1d).

Other factors potentially impacting immune stimulation were also assessed, including age and gender of patients, types of vaccine received and time of sample collection. Moderna mRNA-1273 outperformed Pfizer BNT162b2 vaccine in mean NT_50_ by 2.8-fold for HCWs (p = 0.0053) and 2.1-fold for cancer patients (p = 0.0044) (Fig. 1e). This is consistent with our previous findings that Moderna mRNA-1273 vaccinated individuals exhibit higher NT_50_ levels compared to Pfizer BNT162b2 [5]. Given previous findings that neutralizing antibody response to mRNA vaccination is age dependent [6], we also examined the possible correlation between age and NT_50_ titer. However, no significant correlation between age and NT_50_ values was observed in these cancer patients (Fig. 1f). Notably, while male patients have been shown to exhibit higher NT_50_ levels following COVID-19 disease [7], we found here that female patients in fact exhibited a higher level of virus neutralization with a mean NT_50_ of 299 compared to 154 for males (p = 0.0116; Fig. 1g). This likely reflects an overrepresentation of older patients and patients with hematological cancers in males in our cohort (Table 1).

Given increasing concerns about declining efficacy of SARS-CoV-2 vaccines [8], we also examined the correlation between NT_50_ and time post second vaccine dose for these cancer patients. Indeed, we observed a significant, negative correlation (p = 0.0194) between time after second dose of mRNA vaccination and NT_50_ value (Fig. 1h). These results confirm the waning immune protection of neutralizing antibodies that are conferred by mRNA vaccination.

In summary, by using a sensitive high-throughput lentivirus-based SARS-CoV-2 neutralization assay [3], we have examined the neutralizing antibody response of 160 cancer patients and compared, side by side, with that of 46 healthy HCWs. We observed about an approximately 2.4-fold lower neutralizing antibody response in the cancer patients as compared to HCWs, following Pfizer/BioNTech BNT162b2 or Moderna mRNA-1273 vaccination, clearly demonstrating a reduced efficacy of SARS-CoV-2 spike antibody production among cancer patients. This, along with similar observations of some recent complementary studies [9], should inform the development of novel immunization strategies for cancer patients. In particular, we find that patients with hematological cancers, such as CLL and NHL, are least likely to respond to mRNA vaccination, with 50-60% of these patients showing no detectable levels of neutralizing antibody against the SARS-CoV-2 spike. Given these findings, booster vaccines may be of particular importance for these groups of cancer patients, with some studies already underway [10]. Additionally, our finding that B cell depletion or suppression drug treatment significantly reduced the neutralizing antibody response to mRNA vaccines may indicate a need for immunization to occur during disruptions or suspensions in specific treatment protocols.

Finally, to better protect immunocompromised populations with increased risk to COVID-19, we must further investigate the duration of vaccine induced immunity as well as the efficacy of booster vaccine doses to determine how to maintain protective immunity in this patient population. Additionally, further study on quality and durability of antigen-specific T and memory B cell responses will provide a more comprehensive understanding of the immune response to SARS-CoV-2 vaccination in these immunocompromised groups. It is also critical to determine the impact of specific treatment protocols on vaccine induced immunity and immunity duration to better inform clinical decisions about the time of vaccination or boosting and the potential need for disruptions in treatment protocols. Results from this work provide critical virological and immunological information for protecting vulnerable populations (Additional file 1).

## Supplementary Information

**Additional file 1.** Materials and methods.

## Data Availability

All data and materials will be made available upon requests.

## Abbreviations

CLL: Chronic lymphocytic leukemia
NHL: Non-Hodgkin’s lymphomas
HCW: Health care worker
NT_50_: The 50% neutralizing antibody titer
COVID-19: Coronavirus disease 2019
