# Supplementary material for: Impaired neutralizing antibody response to COVID-19 mRNA vaccines in cancer patients
Source: Cell Biosci. 2021 Nov 21;11:197. doi: 10.1186/s13578-021-00713-2 (PMC8606166; doi:10.1186/s13578-021-00713-2)
Supplement: Supplementary file 1 — Additional file 1. Materials and methods. [file 13578_2021_713_MOESM1_ESM.docx]

**Materials and Methods**

**Vaccinated Cancer Patients and Health Care Workers:**

De-identified vaccinated health care worker (HCW)’s serum samples were collected under approved IRB protocols (2020H0228 and 2020H0527). Serum was collected 6 months after the second dose of Pfizer (n=24) and Moderna (n=22) SARS-CoV-2 mRNA vaccine. The ages of the vaccinated groups ranged from 26 years to 61 years (mean age=38.5).

Cancer patient serum samples were collected under an approved IRB protocol (2021C0041). Serum was collected 31~232 days (median 134 days) after the second dose of Pfizer (n=98) and Moderna (n=62) SARS-CoV-2 mRNA vaccines. The age of vaccinated cancer patients ranged from 31 years to 81 years (mean age=66). These cancer patients consisted of 29 lung cancer, 54 chronic lymphocytic leukemia (CLL), 30 breast cancer, 2 CLL and breast cancer, and 45 various non-Hodgkin’s lymphomas. Patients were 47% (n=75) female and 53% (n=85) male. Twenty-eight of the 54 CLL patients and 19 of the 45 non-Hodgkin’s lymphomas patients received anti-B cell therapy, with drugs including BTK inhibitors and anti-CD20 mAbs during the study period. Twenty-six of the 29 lung cancer patients and 2 of the 30 breast cancer patients received anti-PD-1/PD-L1 therapy.

**Cell Culture:**

HEK293T cells (ATCC CRL-11268, CVCL_1926) and HEK293T-hACE2 cells (BEI NR-52511) were grown in DMEM (Gibco, 11965-092) supplemented with 10% (vol/vol) fetal bovine serum (Sigma, F1051) and 1% penicillin/streptomycin (HyClone, SV30010). Both cell lines were maintained at 37°C, and 5% CO2.

**Constructs:**

The construct used for the production of lentiviral pseudotypes was HIV-1 NL4.3-inGluc[1-3], which was originally obtained from David Derse’s lab at NIH (National Cancer Institute, Frederick, Maryland, USA) and Marc Johnson’s lab at the University of Missouri (Columbia, Missouri, USA). This construct is based on a ΔEnv pNL4.3 HIV-1 vector and contains an anti-sense Gaussia luciferase (Gluc) gene with a sense intron. Gluc is secreted in mammalian cell culture[4], and the intron and anti-sense orientation of the Gluc gene prevents the production of Gluc in the virus producer cells[1-3]. pcDNA3.1-SARS-CoV2-S-C9 encoding SARS-CoV-2 full-length spike was obtained from Fang Li’s lab at the University of Minnesota (St. Paul, Minnesota, USA).

**Virus Production:**

Lentiviral pseudotyped virus was produced as previously described[1]. Briefly, HEK293T cells were transfected with HIV-1 NL4.3-inGluc and pcDNA3.1-SARS-CoV2-S-C9 constructs in a 2:1 ratio using polyelthylenimine (PEI). Supernatants were harvested 24 hr, 48 hr, and 72 hr post-transfection and were pooled, aliquoted, and stored at -80°C.

**Pseudotype Virus Neutralization Assays:**

Pseudotyped virus neutralization assays (VNAs) were performed as previously described[1]. Briefly, cancer patients or vaccinee individual serum was 4-fold serially diluted in 96-well plate (Cellstar, 655180), resulting in a final volume of 60 μL. Subsequently, 100 μL of pseudotyped virus was added to the plate resulting in a final set of dilutions of 1:40, 1:160, 1:640, 1:2560, 1:10240, and no serum. Virus and serum mixture were incubated for 1 hr at 37°C, and then added to HEK293T-ACE2 cells seeded at 2 x 10^4^ cells/well. Media was changed after 6 hrs post-infection. At 48 hrs and 72 hrs after infection, 20 μL of media was collected from the cells and transferred to a white, flat-bottomed, polystyrene 96-well plate (Thermo Scientific, 236108). 20 μL of Gaussia luciferase substrate (0.1 M Tris (MilliporeSigma, #T6066) pH 7.4, 0.3 M sodium ascorbate (Spectrum, S1349), 10 μM coelenterazine (GoldBio, CZ2.5)) was added to the media and immediately read by a BioTek Cytation5 plate-reader.

**Quantification and Statistical Analysis:**

Data were analyzed as mean with Standard Error of Mean (SEM), Statistical analyses were performed using GraphPad Prism 5.0 as follows: One-way Analysis of Variance (ANOVA) with Bonferroni’s post-tests was used to compute statistical significance (p values) between multiple groups for multiple comparison or t-test was used for two groups for single comparison. The 50% neutralization titer (NT_50_) was determined using the half-maximal inhibitory concentration values of plasma samples, normalized to control infection, from their serial dilutions. NT_50_ values were calculated from VNA output using a non-linear regression with least-squares fit in GraphPad Prism5 (GraphPad Software, San Diego, California USA, [www.graphpad.com](http://www.graphpad.com)).

**Supplementary references:**

1. Zeng C, Evans JP, Pearson R, Qu P, Zheng Y-M, Robinson RT, Hall-Stoodley L, Yount J, Pannu S, Mallampalli RK: **Neutralizing antibody against SARS-CoV-2 spike in COVID-19 patients, health care workers, and convalescent plasma donors**. *JCI insight* 2020, **5**(22).

2. Mazurov D, Ilinskaya A, Heidecker G, Lloyd P, Derse D: **Quantitative comparison of HTLV-1 and HIV-1 cell-to-cell infection with new replication dependent vectors**. *PLoS Pathog* 2010, **6**(2):e1000788.

3. Yu J, Li M, Wilkins J, Ding S, Swartz TH, Esposito AM, Zheng Y-M, Freed EO, Liang C, Chen BK: **IFITM proteins restrict HIV-1 infection by antagonizing the envelope glycoprotein**. *Cell reports* 2015, **13**(1):145-156.

4. Goerke AR, Loening AM, Gambhir SS, Swartz JR: **Cell-free metabolic engineering promotes high-level production of bioactive Gaussia princeps luciferase**. *Metabolic engineering* 2008, **10**(3-4):187-200.
